# Mechanisms of Key Performance Degradation in Silicone Rubber Polymer Insulation for High-Voltage Composite Bushings Under Coupled Temperature, Humidity, and Corona Aging

**DOI:** 10.3390/polym18080935

**Published:** 2026-04-10

**Authors:** Xinhan Qiao, Wentian Zeng, Wenyu Ye, Xize Dai, Jianwen Zhang, Yue Ming

**Affiliations:** 1 School of Electrical Engineering, China University of Mining and Technology, Xuzhou 221116, China; qiaoxinhan@foxmail.com (X.Q.); zengwentian023@163.com (W.Z.); jwzhang@cumt.edu.cn (J.Z.); 2 School of Electrical Engineering and Computer Science, University of Queensland, Brisbane, QLD 4072, Australia; xize.dai@uq.edu.au; 3 State Key Laboratory of Power Transmission Equipment Technology, School of Electrical Engineering, Chongqing University, Chongqing 400044, China; 4 School of Mechanical & Electrical Engineering, Xuzhou University of Technology, Xuzhou 221018, China

**Keywords:** high-voltage bushing, silicone rubber, aging test, hydrophobic property, mechanism research, corona

## Abstract

To investigate the multi-factor aging mechanisms of silicone rubber used in the outer sheath of composite bushings, this study focused on HTV silicone rubber employed in the sheath layer of 1100 kV high-voltage bushings. The samples were subjected to temperature–humidity–corona coupled aging in a multi-factor aging platform. The aged samples were characterized by scanning electron microscopy, energy-dispersive spectroscopy, Fourier-transform infrared spectroscopy, hydrophobicity measurements, hardness tests, and dielectric constant measurements. The results indicate that different aging factors affect the material differently. Corona aging primarily affects the sample surface, leading to substantial methyl group detachment, surface oxidation, and a decrease in hydrophobicity, with the local static contact angle decreasing by up to 70%. In contrast, wet heat aging affects the bulk material; under high-temperature and high-humidity conditions, the internal small-molecule chains accelerate silicon-oxide crosslinking, leading to a marked increase in hardness and a relative dielectric constant that initially decreases and then increases. Considering the complex field environment, surface performance measurements are easily influenced by external factors. Therefore, hardness and relative dielectric constant are proposed as key indicators for evaluating the aging degree of silicone rubber sheaths in service. The findings provide a valuable reference for the service-life evaluation of composite bushings.

## 1. Introduction

Composite bushings are widely employed across various voltage levels in power systems due to their excellent insulation performance, high resistance to temperature variations [1], and lightweight structure [2,3,4]. However, with prolonged service, many bushings in power grids undergo aging, and the extent of degradation is often difficult to quantify. They are frequently replaced only after failures occur, which poses potential risks to grid reliability and operational safety [5,6]. However, research on the aging life of composite bushings remains a critical topic, and the mechanisms of the key factors contributing to bushing aging warrant further investigation. High-voltage composite bushings typically comprise flanges, conductive rods, shielding layers, insulating sleeves, and silicone rubber sheaths [7]. The sheath serves as the outer insulation and protective layer, being directly exposed to environmental stresses, and is therefore the component most susceptible to degradation [8]. Aging of the silicone rubber sheath can result in insulation deterioration and moisture ingress, substantially reducing the service life of high-voltage bushings [9,10]. Accordingly, investigating the aging mechanisms of the sheath layer is critical for ensuring the long-term reliability of high-voltage bushings.

Silicone rubber is the primary material used in composite bushing sheaths. From a manufacturing perspective, it can be classified into high-temperature vulcanized (HTV), room-temperature vulcanized (RTV), and liquid silicone rubber (LSR) [11]. Compared with LSR, HTV silicone rubber exhibits superior high-temperature resistance and mechanical properties, making it the preferred choice for ultra-high-voltage applications [12,13]. During service, the sheath layer is exposed to temperature, humidity, and strong electric fields, which can induce surface cracking and powdering, leading to a loss of hydrophobicity, reduced mechanical strength, and accelerated aging under distorted electric fields [14,15]. Research on the aging characteristics of silicone rubber for bushings primarily follows two approaches. The first approach involves the analysis of in-service or retired samples. For example, Enciso et al. conducted physical and chemical tests on retired high-voltage bushings and summarized early failure patterns [16]. Wang et al. investigated the aging of silicone rubber in 500 kV current transformer bushings and reported that aging is mainly associated with Si–C bond breakage and excessive crosslinking of the polymer backbone [17]. Mu et al. examined detached silicone rubber layers from both material and mechanical perspectives and concluded that performance degradation is primarily related to oxidation and over-crosslinking [18]. The second approach involves accelerated aging tests on new samples to elucidate material aging behaviors. Zeng et al. performed ultraviolet–mechanical aging experiments on HTV silicone rubber, demonstrating that UV exposure promotes surface crosslinking [19]. Kashi et al. exposed silicone rubber to polyalkylene glycol at elevated temperatures and observed significant deterioration of mechanical properties under thermal stress [14]. Venkatesulu et al. investigated corona discharge aging using a needle-plate electrode and found that the addition of nano-fillers substantially enhanced anti-corona performance [20].

Compared with field sampling, artificial accelerated aging enables precise control of individual variables, facilitating the independent investigation of single-factor aging mechanisms and reducing experimental duration [14]. However, the existing literature has predominantly focused on single- or dual-factor aging under thermal and humid conditions, with limited research dedicated to multi-factor coupling [21,22,23]. Reference [21] investigated multi-factor aging incorporating temperature cycling, elucidating the degradation mechanisms of HTV silicone rubber under such conditions. Building upon this, Reference [24] introduced humidity gradients to further explore the coupled mechanisms of temperature, humidity, and corona discharge. Nevertheless, corona discharge characteristics are inherently influenced by temperature and humidity; consequently, the aging intensity experienced by samples fluctuates significantly under temperature cycling, which complicates the decoupling of individual factor effects.

To further investigate the coupled mechanisms of temperature–humidity–corona, this study conducts multi-factor aging experiments on HTV silicone rubber used in ultra-high voltage bushings, employing varied temperature, humidity, and time gradients. The aging characteristics of the HTV silicone rubber were systematically analyzed using scanning electron microscopy, energy-dispersive spectroscopy, Fourier-transform infrared spectroscopy, hydrophobicity evaluation, hardness testing, and dielectric property measurements. The results indicate that different aging factors affect the material differently. Corona aging primarily affects the sample surface, leading to substantial methyl group detachment, surface oxidation, and a decrease in hydrophobicity, with the local static contact angle decreasing by up to 70%. In contrast, wet heat aging affects the bulk material; under high-temperature and high-humidity conditions, the internal small-molecule chains accelerate silicon-oxide crosslinking, leading to a marked increase in hardness and a relative dielectric constant that initially decreases and then increases. The findings provide a valuable reference for the service-life evaluation of composite bushings.

## 2. Experimental Setup

### 2.1. Experimental Samples and Experimental Platforms

The silicone rubber samples used in this study were sourced from Jiangsu Shema Electric Co., Ltd. (Nantong, China) for the production of high-temperature vulcanized silicone rubber materials for the outer sheath of 1100 kV composite sleeves. The sample was primarily composed of siloxane, with silica serving as a reinforcing agent and aluminum oxide as a flame retardant. Additionally, tungsten-containing compounds and other additives were incorporated to enhance the mechanical properties. To facilitate the experiment, the high-temperature vulcanized silicone rubber was shaped into a square sheet with a side length of 10 cm and a thickness of 2 mm. Before the experiment, the sample surface was cleaned with 95% alcohol to minimize the impact of surface contamination on the sample. After the alcohol evaporated and dried, the sample was placed on a copper plate beneath the multi-needle plate electrode, with an unaged sample placed beside it for comparative analysis.

**Figure 1:**
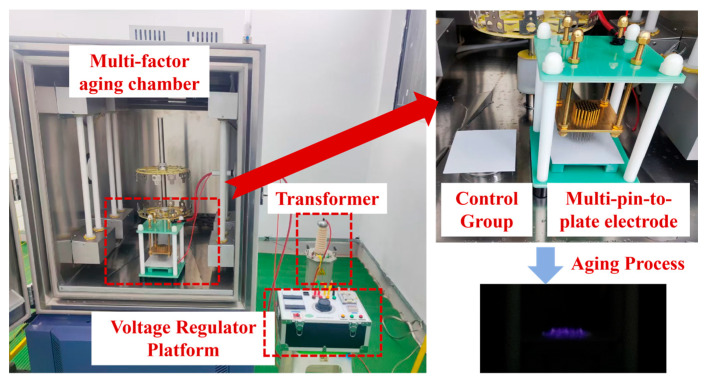
Multi-factor aging platform.

The aging experimental platform consists of a multi-factor aging chamber, multi-pin-plate electrodes, a transformer, a voltage-regulating table, grounding rods, and a safety fence. The overall platform is shown in Figure 1. Among them, the multi-pin-plate electrodes are placed inside the aging chamber. The high-voltage end of them passes through a sleeve and is connected to the high-voltage output end of the transformer, while the low-voltage end is led out for grounding through a sleeve. The aging factors are mainly provided by the aging chamber and the multi-pin-plate electrodes. The multi-factor aging chamber is responsible for controlling the temperature and humidity environment where the samples are placed, and the multi-pin-plate electrodes are used to generate corona on the surface of the samples to simulate a strong electric field environment.

In the upper right corner of Figure 1, the multi-pin plate electrode device is shown. The copper plate on top is the high-voltage end and is connected to a multi-pin array. The copper plate at the bottom is the low-voltage end. After testing, at voltages higher than 5 kV, the needle tips of the multi-pin-plate electrode will emit current sounds and exhibit discharge phenomena. At around 10 kV, the corona phenomenon is more obvious, and clear discharge phenomena can be observed. Therefore, the corona voltage was set at 10 kV.

The experiment was performed using different temperatures, humidities, corona conditions, and aging times to explore the aging characteristics of the silicone rubber under different factors. To comprehensively investigate the mechanism of temperature, humidity, corona, and aging time on silicone rubber, it is necessary to balance the intensity of the effects of temperature, humidity, and corona as much as possible. Due to the significant effect of the corona, in this experiment, the temperature and humidity were set to the maximum conditions achievable by the aging platform, namely 90 °C and 80% relative humidity, with 60 °C and 40% relative humidity serving as a reference. The specific aging parameters are shown in Table 1.

### 2.2. Sample Performance Testing

After re-treating the aged sample surface with 95% alcohol, various physical and chemical performance tests were conducted for analysis. The test methods are as follows:

(1) Scanning Electron Microscopy Test: Take a 1 cm × 1 cm sample from the sample, and before testing, perform 20 min of ultrasonic cleaning on the sample. Then, conduct gold spraying treatment on the sample surface to enhance conductivity and improve imaging quality. After the treatment, observe it through the TESCAN MIRA scanning electron microscope. The scanning electron microscope was manufactured in Brno, Czech Republic. The experimental process was carried out in accordance with the standard ISO/TS 21383:2021 [25].
(2) Energy Spectrum Test: While observing with the scanning electron microscope, use the QUANTAX electro-optical spectroscopy instrument to scan and analyze the sample surface, obtaining information on the distribution and content of elements on the sample surface. The EDS instrument was manufactured in Berlin, Germany. The test process was carried out in accordance with the standard ISO 22309:2011 [26].
(3) Fourier-Transform Infrared Spectroscopy Test: Use the Thermo Scientific Nicolet™ iS50 type spectrometer to scan the sample surface in the 400–4000 cm^−1^ wavelength range. This instrument is manufactured in Madison, Wisconsin, USA. The data resolution was 0.93 wave numbers. A 1 × 1 cm sample was cut from the aged region, and the aged surface was subjected to testing. The sample surface was continuously scanned 32 times, and the final result was obtained by signal averaging. The test process was carried out in accordance with the standard ISO 11339:2022 [27].
(4) Hydrophobicity Test: Due to the hydrophobic recovery property of the silicone rubber material, after the material’s hydrophobicity stabilizes (i.e., 24 h after the aging process ends), the hydrophobic performance of the sample surface was tested. The hydrophobic performance was measured using the static contact angle method. Using a pipette, 2 μL of pure water droplets were dropped onto the sample surface, and the angle between the water droplet and the sample surface was captured by a camera. Finally, the static contact angle of the water droplet in the image was measured. The experiment was conducted at multiple aging positions, and the results were recorded. The specific process of the experiment followed the standard ISO 15989:2023 [28].
(5) Hardness Test: Using a Shore A hardness tester, hardness measurements were taken at 8 random locations on the aged samples. The average value was taken as the result. The test process followed the standard ISO 7619-1:2010 [29]. In compliance with the standard, specimens thinner than 6 mm may be superimposed to achieve the required thickness. Consequently, three samples for each working condition were aged and subsequently stacked for the final test.
(6) Dielectric Constant Test: The sample was tested using an Agilent 4294A impedance analyzer. This instrument is manufactured in Santa Clara, CA, USA. Before the test, the upper and lower surfaces of the sample were silver-plated. Then, 20 points were randomly selected from each sample to test the dielectric curve of the sample at room temperature and frequencies ranging from 40 to 106 Hz. The average value of the results was taken. The test process followed the standard IEC 60250:1969 [30].

## 3. Analysis of Experimental Results

### 3.1. Macroscopic Morphological Analysis

**Figure 2:**
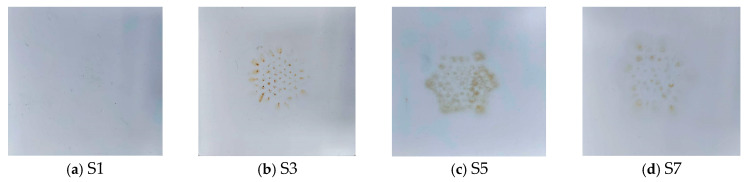
Surface morphology of the corona aging sample.

Figure 2 shows the macroscopic surface morphology of the silicone rubber samples after multi-factor aging. Since the macroscopic surface morphology of the samples subjected to humid heat aging showed no significant changes, only the morphology of the samples containing the corona effect is presented.

From Figure 2, it can be seen that after corona aging, there are obvious burn marks on the surface of the samples, and the surface color of the samples in the corona aging range becomes darker. Compared with the first three samples, under the same 36 h temperature and humidity as well as corona aging conditions, their surface morphologies also differ, basically following the rule that the higher the temperature and humidity, the more obvious the aging effect. The increase in temperature will deepen the degree of local aging, and the increase in humidity will expand the aging range. This is probably because the corona effect is also affected by temperature and humidity. When the humidity in the air increases, the discharge effect of corona will be more significant due to the increase in the relative dielectric constant in the gaps, thus resulting in a larger corona range.

### 3.2. Scanning Electron Microscope Testing and Analysis

**Figure 3:**
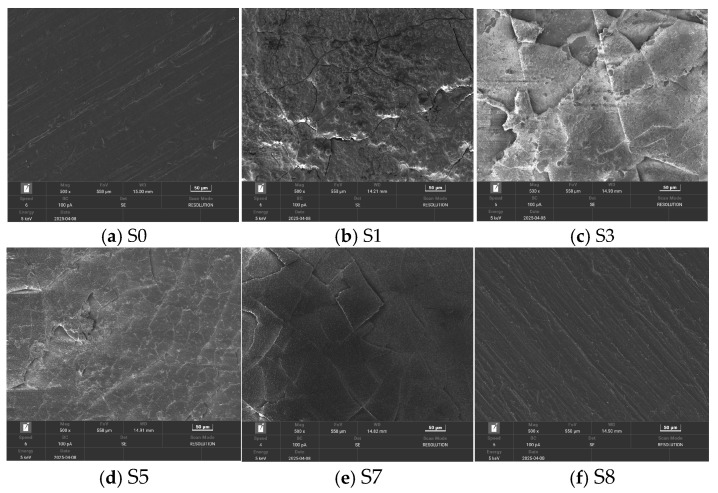
Scanning electron microscope image of the corona aging sample at a magnification of 500 times.

The scanning electron microscope test was conducted on the samples. Since the surface micromorphology of samples without corona aging showed no significant changes, only the unaged sample S0 and the sample subjected to the longest hygrothermal aging S8 are presented here for comparison with the corona-aged samples. Figure 3 shows the images of some samples magnified 500 times.

A comparison between samples S0 and S8 reveals that short-term wet heat aging had minimal effect on the microscopic morphology of the samples, with the overall appearance maintaining a gel-like texture. However, significant aging features were observed on the surfaces of the samples subjected to corona aging. For sample S1, large cracks and numerous spherical protrusions were evident, while the surface appeared relatively intact, with signs of fragmentation about to occur. In sample S3, large cracks and visible detachment of fragments were observed. Sample S5 exhibited fine cracks, with some areas showing signs of detachment, while the unattached regions featured prominent surface protrusions. In sample S7, large fragments had detached, leaving behind areas covered with numerous cracks and smaller spherical protrusions.

**Figure 4:**
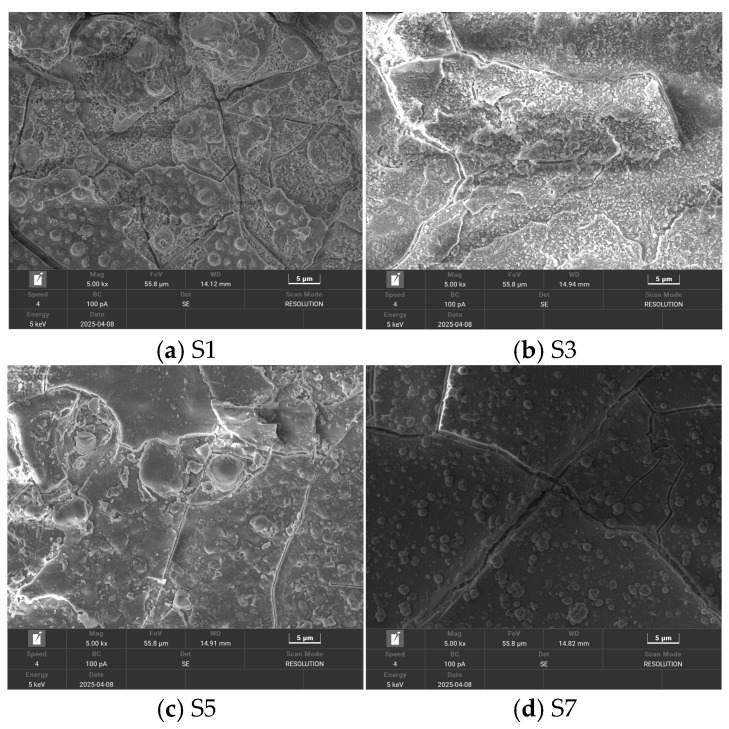
Scanning electron microscope image of the corona aging sample at a magnification of 5000 times.

The samples were further examined at a magnification of 5000× to observe detailed surface features, as shown in Figure 4. Under high magnification, progressive surface erosion is clearly observed with increasing aging severity, accompanied by the formation of numerous small spherical particles. These particles exhibit relatively uniform size and morphology, and no distinct elemental features were detected in the subsequent energy-dispersive spectroscopy analysis. Therefore, these particles are most likely attributed to the aggregation and precipitation of siloxane chains induced by localized high-temperature effects during corona aging.

**Figure 5:**
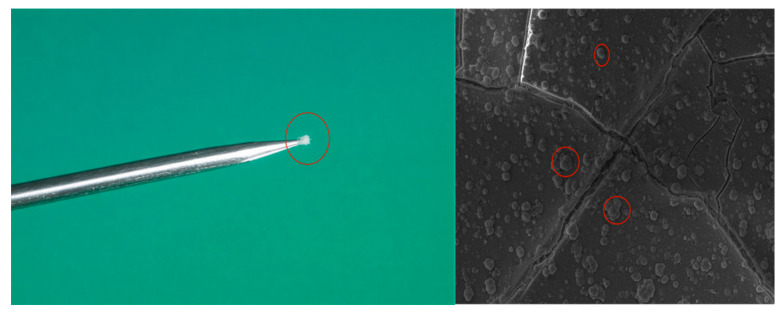
The tip is coated with powder-like particles of silicone rubber.

It is worth noting that after the completion of the corona aging experiment, a large number of fine particles were observed to adhere to the surface of the needle tip, and obvious powdering phenomenon appeared on the surface of the sample, as shown in the red circle in Figure 5. Based on the SEM observations, it can be inferred that these fine particles originated from detached surface fragments and spherical protrusions and were subsequently attracted to the needle tip under the action of the electric field. In practical operation, powdering is frequently observed on the surfaces of silicone rubber sheaths in service. However, this phenomenon has not been clearly reproduced in previous laboratory aging studies of silicone rubber. Since no evidence of siloxane chain aggregation and precipitation was found in the FTIR results presented in Section 3.4, it is hypothesized here that the pulverized particles are primarily caused by the local high-temperature and strong electric field environment. The formation mechanism, composition, and evolution of these powder particles warrant further investigation and will be addressed in future work.

### 3.3. Energy Spectrum Testing Analysis

**Figure 6:**
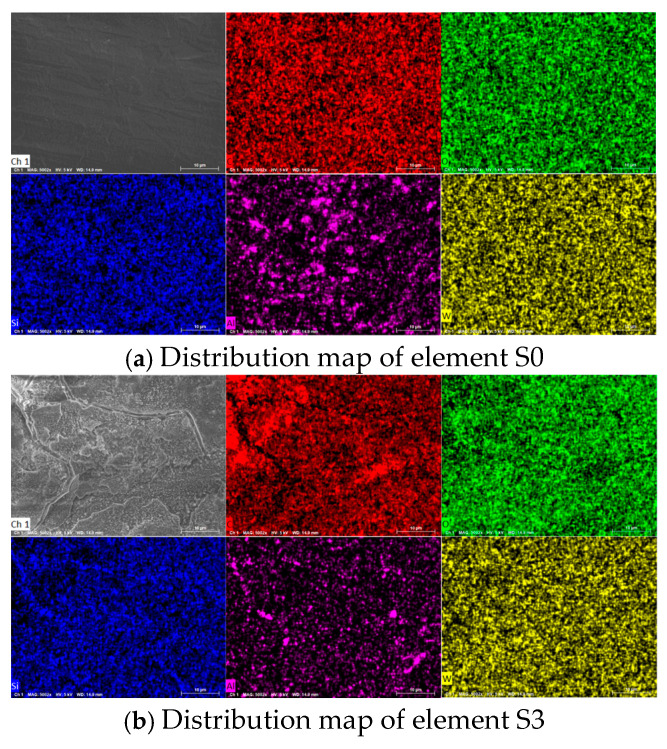

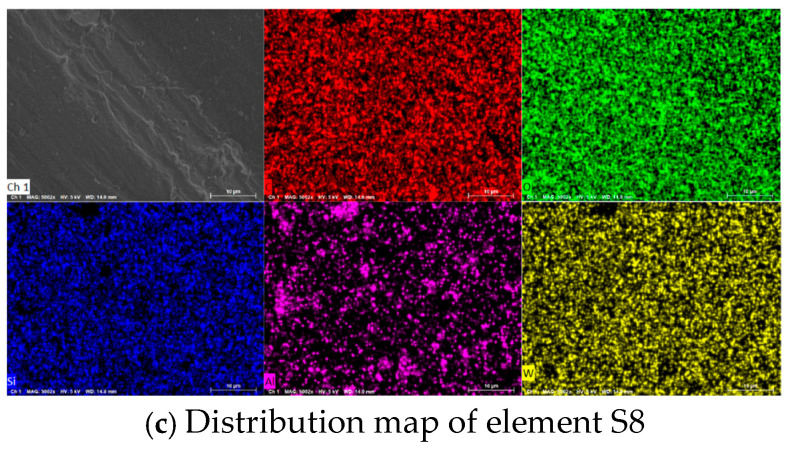
Distribution map of sample elements.

Energy-dispersive spectroscopy (EDS) analysis was performed to obtain the elemental distribution and content information of the aged samples. Samples S0, S3, and S8 were selected to represent the reference, corona-aged, and hygrothermal-aged conditions, respectively. The results are shown in Figure 6.

The elemental distribution analysis reveals that the silicone rubber sample primarily consists of five elements: carbon, oxygen, silicon, aluminum, and tungsten. Tungsten was added as an additive to enhance the mechanical properties and thermal stability of the sample. Given its low content and minimal impact, it is not further analyzed here. The four elements—carbon, oxygen, silicon, and aluminum—are commonly present in HTV silicone rubber. Carbon, oxygen, and silicon form the main backbone of silicone rubber, while aluminum, often introduced as aluminum hydroxide, acts as a flame retardant in HTV silicone rubber [31].

**Figure 7:**
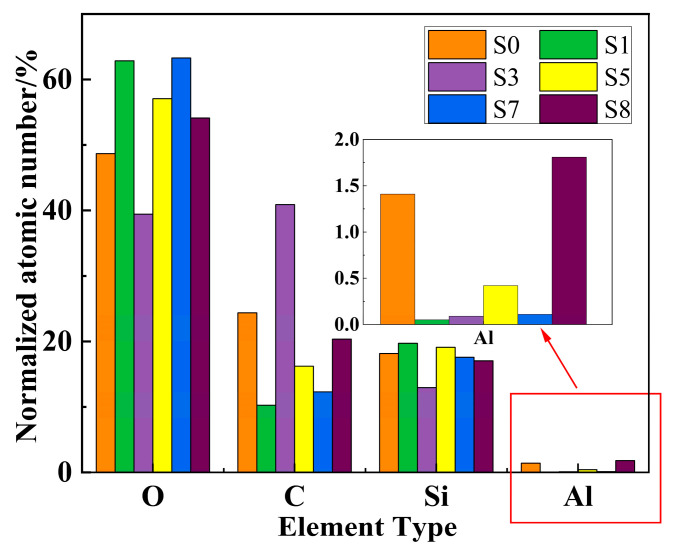
Element content of samples.

As shown in Figure 6, the elemental distribution on the surface of samples without corona aging exhibits no significant variation. In contrast, a pronounced reduction in aluminum content is observed on the surface of sample S3 after corona aging, accompanied by an evident clustering of carbon. The elemental contents of the samples were further quantified, and the statistical results are presented in Figure 7. Based on the variations in elemental composition, the aging-induced surface evolution can be inferred. Samples subjected solely to wet heat aging exhibit an increase in oxygen content and a corresponding decrease in carbon content, which can be attributed to surface oxidation and the detachment of methyl side chains. Under corona exposure, localized high-temperature regions are generated on the sample surface and are repeatedly impacted by arc discharge. In addition, under high-humidity conditions, nitric acid formed in the discharge environment may further corrode the sample surface. Consequently, in high-temperature and high-humidity environments, silicone rubber undergoes more severe surface oxidation, resulting in elevated oxygen content. In contrast, under low-humidity conditions, the corona discharge becomes more concentrated, leading to higher localized temperatures. The intense energy injection promotes dehydrogenation reactions on the surface, accelerating carbonization. As a result, the surface color of the samples gradually darkens, and the carbon content increases.

In silicone rubber, aluminum typically exists as aluminum hydroxide, which acts as a flame retardant. Under high-temperature conditions, aluminum hydroxide decomposes into aluminum oxide while releasing water, helping to dissipate heat. However, after corona aging, the content of aluminum elements significantly decreases and becomes nearly undetectable. This is likely due to the weakening of the bond between aluminum oxide and the surrounding silicon oxides under the influence of the corona effect, which may lead to the transformation of aluminum oxide into independent powder particles. These particles are subsequently adsorbed onto the needle tip.

### 3.4. Fourier-Transform Infrared Test Results

Fourier-transform infrared tests were conducted on S0, S1, S3, S5, S7, and S8, respectively. Due to the strong recovery property of the silicone rubber samples after aging, the surface groups would undergo significant changes within a short period of time. Therefore, the Fourier-transform infrared test was carried out on the surface of the samples after a certain period of time had elapsed since the aging of the samples. At this time, the surface groups of the samples were relatively stable, and the differences in the steady state after aging of the samples could be observed.

The transmittance in the figure represents the residual rate of infrared light passing through the sample in a specific wavelength band. The smaller the transmittance, the more the sample absorbs the light in that wavelength band, that is, the more the corresponding groups. For silicone rubber, we mainly focused on the methyl groups at 2960 cm^−1^, 1260 cm^−1^, and 788 cm^−1^, as well as the silicon–oxygen bond at 1010 cm^−1^. The methyl group, as a side chain of the siloxane molecule, mainly plays a hydrophobic role and reflects the hydrophobic property of the silicone rubber; the silicon–oxygen bond is the main chain of the silicone rubber molecule, and its increase or decrease mainly reflects the mechanical properties of the silicone rubber. The specific numerical values are shown in Table 2.

From the table, it can be seen that both the methyl groups and the siloxanes in the aged samples have significantly decreased. By combining the data at 2960 cm^−1^, 1260 cm^−1^, and 788 cm^−1^, the order of methyl loss from most to least is S7, S3, S5, S1, and S8. Therefore, it can be concluded that under the coupled stress of high temperature, high humidity, and corona discharge, the degradation of surface methyl groups is predominantly governed by the corona effect. The direct impact of temperature and humidity is minimal; instead, they influence the methyl groups indirectly by modulating the corona discharge intensity. Notably, humidity exerts a more significant influence on the corona process than temperature.

The loss of silicon–oxygen bonds at 1010 cm^−1^ is from high to low: S7, S1, S3, S5, and S8. A comparison between S8 and S0 indicates that short-term thermal aging under humid conditions has a relatively minor effect on the silicon–oxygen main chain. Under corona discharge, temperature exerts a greater influence than humidity. This may be attributed to the higher bonding strength of the silicon–oxygen bonds, which reduces the susceptibility of the chemical bonds to electron bombardment. During corona discharge, the elevated local temperature has a more pronounced effect, whereas in high-humidity environments, water absorbs part of the thermal energy, thereby mitigating the damage to the silicon–oxygen bonds.

Notably, a new characteristic peak appears at 1640 cm^−1^ in the corona-aged samples. This band is typically associated with adsorbed water (H–O–H bending vibration). The emergence of this peak provides indirect evidence that local surface hydrophobicity decreases after corona aging, allowing adsorption of water molecules during the measurement.

**Figure 8:**
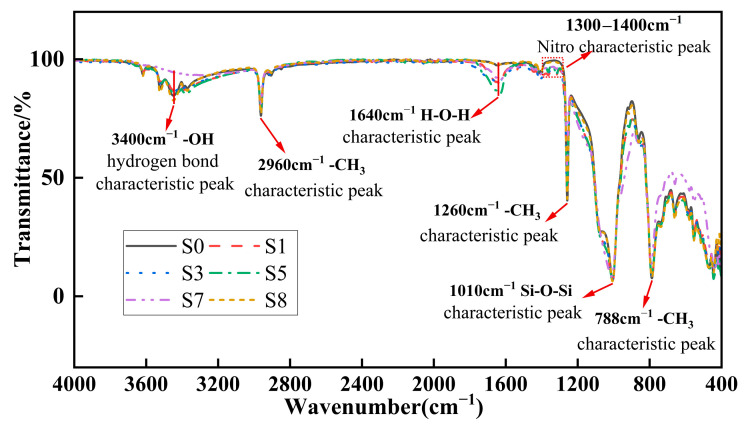
Fourier-transform infrared test waveform.

Figure 8 presents the overlap of the six FTIR spectra. In addition to the five characteristic peaks summarized in Table 2, differences are observed in the hydroxyl peak at 3400 cm^−1^ and the nitro group region at 1300–1400 cm^−1^ between corona-aged samples and those without corona aging. After corona aging, the surface hydroxyl content decreased significantly, although a peak corresponding to adsorbed or bound water was still observed at 3400 cm^−1^. To elucidate this, the contribution of adsorbed and bound water should be excluded by considering the peak at 1640 cm^−1^. As the intensity of the 1640 cm^−1^ peak increases after corona aging, while the total hydroxyl signal at 3400 cm^−1^ decreases, it can be concluded that the actual surface hydroxyl content decreases. Furthermore, the appearance of a peak in the 1300–1400 cm^−1^ region suggests the formation of nitro groups. This may be attributed to the oxidation of surface hydroxyl groups by nitric acid generated during corona aging, leading to water removal and replacement of the original hydroxyl sites with nitro functionalities. Meanwhile, since the pyrolysis temperature of ATH ranges from 200 to 300 °C, the local high temperatures induced by corona aging can cause the aluminum hydroxide in ATH to decompose into alumina and water. This decomposition is also one of the reasons for the reduction of hydroxyl groups at 3400 cm^−1^.

### 3.5. Hydrophobicity Analysis

The presence of water droplets on the cable sheath surface can distort the electric field, increasing the local electric field intensity and accelerating aging, potentially leading to insulation failure. Therefore, the change in hydrophobic properties during the aging process of the sheath material is of particular concern.

Due to the non-uniform nature of corona aging, the worst hydrophobic performance data obtained from each sample test were compared. The results are presented in Table 3. It is evident from the table that corona aging significantly deteriorates the hydrophobicity of the sample surface. The most severely aged sample, S3, exhibited a static contact angle of 32.92°, indicating a complete loss of hydrophobic properties. A comparison between S1, S5 and S2, S6 reveals that the loss of hydrophobicity is positively correlated with environmental temperature. Similarly, comparing S3, S5 with S4, S6 shows that the loss of hydrophobicity is negatively correlated with environmental humidity. Finally, comparing S5, S7 with S6, S8, it is observed that hydrophobicity is positively correlated with aging time.

**Figure 9:**
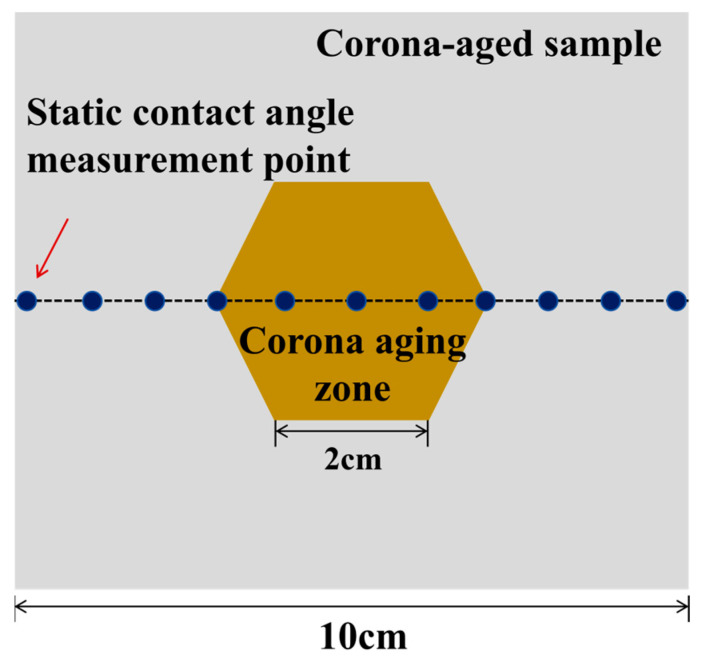
Diagram of water repellency distribution measurement.

Due to the non-uniformity of corona aging, subsequent measurements were conducted on S1, S3, S5, and S7 that had undergone corona aging using several measurement points, as shown in Figure 9. The points were spaced 1 cm apart to observe the overall hydrophobicity distribution of the sample.

**Figure 10:**
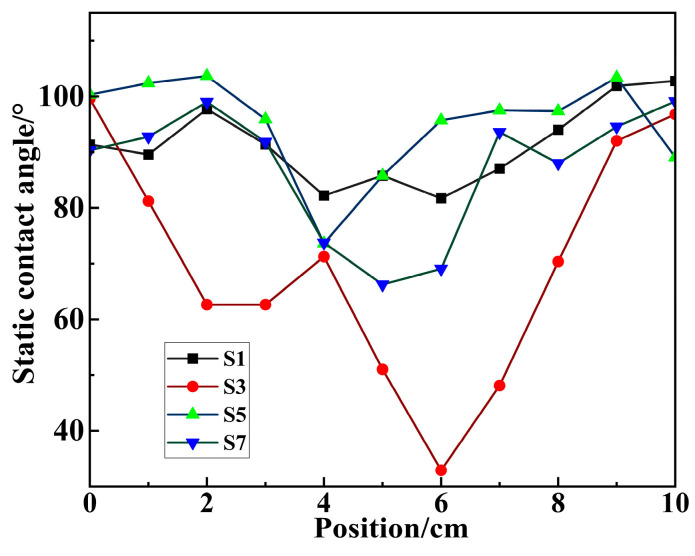
Static contact angle distribution of corona aging samples.

The static contact angle distribution for the four samples along the horizontal axis, with the leftmost measurement point as the origin, is shown in Figure 10. By comparing S3 with the other samples, it is evident that corona discharge causes more severe damage to hydrophobicity in low-humidity environments. Within 2 cm of the corona aging zone, hydrophobicity is significantly affected. For the other samples, the hydrophobicity remains relatively stable until they enter the aging zone. Furthermore, significant variations in hydrophobicity are observed within the same sample, with a general trend of increased degradation with higher temperature, lower humidity, and longer aging time. The most severe hydrophobicity loss occurs within a 1 cm radius from the center of the aging zone, where the electric field intensity is also highest.

### 3.6. Hardness Testing Analysis

The hardness of nine samples was tested, with the corona-aged samples divided into two regions, namely the corona aging zone and the non-corona aging zone, to assess the impact of corona discharge on hardness. The test results are presented in Table 4. From the table, it is evident that temperature, humidity, corona exposure, and aging time all show a positive correlation with hardness. Unlike the significant impact of corona on hydrophobicity, the effect of corona on hardness is relatively less pronounced. This suggests that corona primarily affects the surface properties of the sample, whereas hardness is more closely related to the overall material properties. Consequently, the effect of corona on sample hardness is not as significant as its effect on hydrophobicity.

### 3.7. Dielectric Constant Test

**Figure 11:**
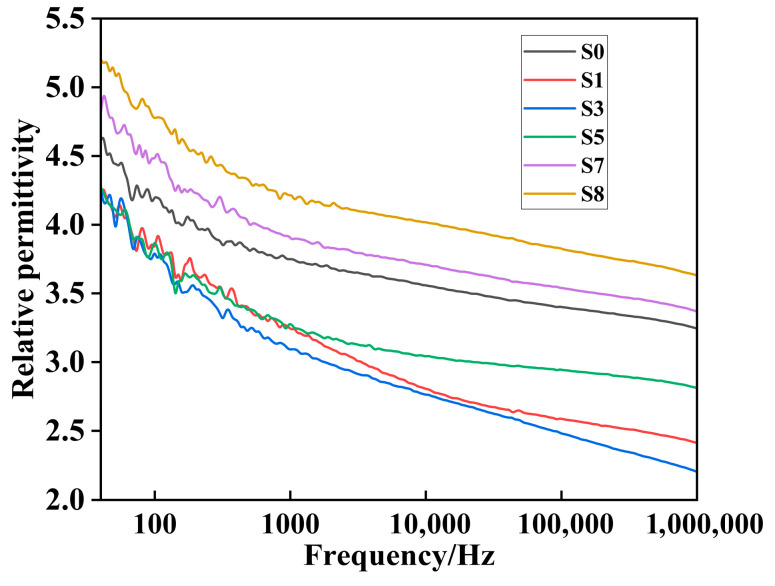
Relative permittivity–frequency curve.

The relative dielectric constants of the six main samples were measured, and the results are shown in Figure 11. As observed in the figure, the relative dielectric constants of the samples decrease with increasing frequency. This behavior can be attributed to the nature of silicone rubber as a polymer. Under an alternating electric field, silicone rubber primarily experiences dipole polarization. As the frequency of the applied electric field increases, the rotation of the dipoles becomes slower than the frequency of the electric field oscillations, leading to a decrease in the relative dielectric constant.

The relative dielectric constants of the six samples are presented in Table 5. At frequencies of 50 Hz and 10^6^ Hz, the relative dielectric constants, ranked from high to low, are S8, S7, S0, S5, S1, and S3. The relative dielectric constant is primarily influenced by moisture content and free radicals within the samples, which have two main effects. On the one hand, as electrothermal aging progresses, the crosslinking degree of the silicone rubber short chains increases, making overall polarization more difficult, which leads to a decrease in the relative dielectric constant. On the other hand, as aging progresses, the hydrophobic methyl groups decrease, allowing more moisture to be adsorbed onto and into the samples. Given the relatively high dielectric constant of water (81), this results in an increase in the overall relative dielectric constant.

During the early stages of aging, the crosslinking effect outweighs the moisture invasion, causing the relative dielectric constant to decrease. However, as aging continues, the crosslinking process stabilizes, and moisture invasion becomes more dominant, leading to an increase in the relative dielectric constant.

## 4. Discussion

Based on the results of the above experiments, the effects of corona discharge, humidity, and heat on silicone rubber can be classified into two categories: surface effects and overall effects. The magnitudes of these effects differ between the two categories.

### 4.1. The Influence of Temperature, Humidity, and Corona Aging on the Physical and Chemical Properties of Silicone Rubber Surfaces

Based on the results from scanning electron microscopy, energy-dispersive spectroscopy, Fourier-transform infrared spectroscopy, and hydrophobicity testing, it can be concluded that the combined effects of corona discharge, high humidity, and heat lead to the most significant damage through corona aging. Even after only 36 h of aging, the sample surface experiences substantial deterioration. A significant number of methyl groups detach from the surface, causing a considerable loss of local hydrophobicity. Simultaneously, the ablation effect of corona discharge causes the sample surface to harden, transitioning from a gel-like state to carbonization. The surface loses elasticity and becomes unevenly abraded under corona discharge, leading to the detachment of fine silicone rubber fragments.

After 72 h of corona aging, clear ablation damage marks are visible on the sample surface. Thus, within the timescale of corona aging, the effect of wet heat aging on the surface is minimal. Typically, the wet heat environment first influences the output of corona discharge, after which the corona discharge directly impacts the sample surface.

### 4.2. The Influence of Temperature, Humidity, and Corona Aging on the Overall Physical and Chemical Properties of Silicone Rubber

Based on the results from both hardness and dielectric tests, it can be concluded that wet heat aging primarily influences the overall aging of the sample, with temperature having a more significant effect on hardness and humidity having a greater impact on dielectric properties. In the same wet heat environment, the hardness of the corona-aged samples showed only a slight increase compared to the non-corona-aged samples. This suggests that corona discharge mainly affects the surface of the sample, with minimal impact on the deeper aging of the silicone rubber. The relative dielectric constant of the sample is also significantly influenced by wet heat, showing a trend of first decreasing and then increasing as aging progresses.

Since the sample surface is easily contaminated by the external environment and affected by the movement of small-molecule silicone oxides, surface performance test results are often inconsistent with the overall aging degree, making it difficult to accurately assess aging based solely on surface tests. However, changes in hardness and dielectric properties are more stable. For instance, in terms of hardness, higher temperatures and longer aging times lead to a greater increase in sample hardness, with this change accumulating steadily over time. In terms of dielectric properties, the dielectric constant decreases in the early stages of aging and increases at higher aging states. Combining hardness and dielectric constant provides a reliable method for assessing the aging degree of the composite bushing sheath layer.

## 5. Conclusions

This paper presents an accelerated aging experiment on the silicone rubber material of the casing sheath under the coupled conditions of temperature, humidity, and corona discharge. The surface microstructure, elemental distribution, group content, hydrophobicity, hardness, and dielectric constant of the aged samples were tested. The main conclusions are as follows:

(1) In a corona environment, the aging effect due to corona discharge is the most significant. Temperature and humidity primarily influence the output of corona discharge, which in turn affects the aging of the samples. An increase in temperature intensifies the corona aging effect, while increased humidity expands the range of corona aging.

(2) In the early stages of aging, the oxidation of the silicone rubber surface increases, accompanied by the detachment of methyl groups and a decrease in hydrophobicity. The high temperature causes further crosslinking of the internal silicon–oxygen chains, leading to a decrease in dielectric constant and an increase in overall hardness. The hardened surface exhibits fragmentation and cracking under the influence of corona discharge. In the later stages of corona aging, dehydrogenation reactions and gradual carbonization occur, with the sample surface becoming enriched in carbon elements. The color deepens, and powder-like substances appear on the surface. Due to the decrease in hydrophobicity and the infiltration of water molecules, the dielectric constant of the sample increases.

(3) Most surface performance test results are susceptible to external factors and the movement of small molecules within the sample, making it difficult to accurately assess the aging state based on surface tests alone. However, hardness and dielectric properties, reflecting the overall material performance, exhibit more consistent changes during aging, making them reliable indicators for determining the aging state of the sample.

## Figures and Tables

**Table 1:** Aging experiment parameters.

Number	Temperature	Humidity	Corona Aging	Aging Time
S0	Unaged			
S1	60	80	10 kV	36 h
S2	60	80	\	36 h
S3	90	40	10 kV	36 h
S4	90	40	\	36 h
S5	90	80	10 kV	36 h
S6	90	80	\	36 h
S7	90	80	10 kV	72 h
S8	90	80	\	72 h

**Table 2:** Transmittance of key characteristic peaks of the sample.

Number	2960 cm^−1^ -CH_3_	1640 cm^−1^ H-O-H	1260 cm^−1^ -CH_3_	1010 cm^−1^ -Si-O-Si-	788 cm^−1^ -CH_3_
S0	76.19	97.45	40.41	6.38	7.64
S1	79.10	90.01	43.53	6.25	8.56
S3	78.21	90.37	42.91	6.72	9.03
S5	78.90	85.57	45.61	6.64	9.41
S7	81.32	91.46	55.63	8.39	13.70
S8	76.89	97.77	41.11	6.38	7.97

**Table 3:** The static contact angle measurement results of the sample.

Number	S0	S1	S2	S3	S4	S5	S6	S7	S8
Static contact angle/°	112.05	81.71	111.59	32.92	95.68	73.65	109.79	66.24	103.96

**Table 4:** Test results of sample hardness.

Number	Non-Corona Zone	Corona Zone
S0	70.56	/
S1	71.25	71.87
S2	70.87	/
S3	72.00	72.50
S4	71.68	/
S5	73.00	73.37
S6	73.12	/
S7	73.81	74.37
S8	74.00	/

**Table 5:** Relative dielectric constant of the sample at power frequency.

Number	Relative Dielectric Constant at 50 Hz	Relative Dielectric Constant at 10^6^ Hz
S0	4.4487	3.2437
S1	4.0154	2.4125
S3	3.9064	2.2025
S5	4.107	2.8121
S7	4.656	3.368
S8	5.0453	3.6289

## Data Availability

The original contributions presented in this study are included in the article. Further inquiries can be directed to the corresponding authors.

## References

[1] Wang S., Hou M., Ma K., Li Z., Geng H., Zhang W., Li N. (2022). Research on the influence of extremely cold environment on the performance of silicone rubber and fluorinated silicone rubber. Polymers.

[2] Gilak Hakimabadi S., Ehsani M., Esfandeh M. (2024). Polymeric composites and hybrids for high-voltage insulators. Polym.-Plast. Technol. Mater..

[3] Abd-Rahman R., Haddad A., Harid N., Griffiths H. (2012). Stress control on polymeric outdoor insulators using zinc oxide microvaristor composites. IEEE Trans. Dielectr. Electr. Insul..

[4] Liu S., Liu S., Wang Q., Zuo Z., Wei L., Chen Z., Liang X. (2023). Improving surface performance of silicone rubber for composite insulators by multifunctional nano-coating. Chem. Eng. J..

[5] Sit K., Pradhan A.K., Chatterjee B., Dalai S. (2022). A review on characteristics and assessment techniques of high-voltage silicone rubber insulators. IEEE Trans. Dielectr. Electr. Insul..

[6] Song W., Shen W.W., Zhang G.J., Song B.P., Lang Y., Su G.Q., Mu H.B., Deng J.B. (2015). Aging characterization of high-temperature vulcanized silicone rubber housing material used for outdoor insulation. IEEE Trans. Dielectr. Electr. Insul..

[7] Zhou X., Tian T., Dai L., Bai J., Yang W., Luo Y., Pan L., Yu J., Li J., Chang X. (2025). Research on fatigue characteristics of conductive rod of high-pressure bushing under different factors. AIP Adv..

[8] Liang X.D., Li S.H., Gao Y.F., Su Z.Y., Zhou J. (2020). Improving the outdoor insulation performance of Chinese EHV and UHV AC and DC overhead transmission lines. IEEE Electr. Insul. Mag..

[9] Zeng Z., Guo P., Zhang R.S., Zhao Z.R., Bao J.K., Wang Q., Xu Z. (2023). Review of aging evaluation methods for silicone rubber composite insulators. Polymers.

[10] Akbar M., Ullah R., Alam S. (2019). Aging of silicone rubber-based composite insulators under multi-stressed conditions: An overview. Mater. Res. Express.

[11] Wei X., Lu W., Zhang J., Li J., Xu C., Jia Z. Aging characteristics and repairing evaluation of long-term operating LSR bushings. Proceedings of the 2nd International Conference on Electrical Materials and Power Equipment (ICEMPE).

[12] Xu C., Guan R., Jia Z., Wang X., Deng Y. (2022). Energy balance model for providing mechanistic insights into HTV and LSR performance in inclined plane tests. IEEE Trans. Dielectr. Electr. Insul..

[13] Verma A.R., Reddy B.S., Chakraborty R. (2018). Multistress aging studies on polymeric insulators. IEEE Trans. Dielectr. Electr. Insul..

[14] Kashi S., Varley R., De Souza M., Al-Assafi S., Di Pietro A., de Lavigne C., Fox B. (2018). Mechanical, thermal, and morphological behavior of silicone rubber during accelerated aging. Polym.-Plast. Technol. Eng..

[15] Liang T., Zhang Z.J., Jiang X.L., Hu J.L., Hu Q. (2024). A method for simulating powdering of silicone rubber composite insulators in coastal areas. High Volt..

[16] Enciso L., Alvarez R., Martinez J.L., Arce P., Galliani M., Rodriguez P., Calo E. Incipient failures analysis of high-voltage bushings. Proceedings of the IEEE Electrical Insulation Conference (EIC).

[17] Wang Z., Zhang X., Wang F., Lan X., Zhou Y. (2016). Effects of aging on the structural, mechanical, and thermal properties of the silicone rubber current transformer insulation bushing for a 500 kV substation. SpringerPlus.

[18] Mu L., Wang B., Hao J.P., Fang Z.Y., Wang Y. (2023). Study on material and mechanical characteristics of silicone rubber shed of field-aged 110 kV composite insulators. Sci. Rep..

[19] Zeng S.Y., Li W.D., He W.J., Liu Y.L., Yan X.Y., Lu M., Gao C., Zhang G.J. (2025). Effects of combined UV–tensile aging on structural and electrical properties of high-temperature vulcanized silicone rubber in composite insulators. RSC Adv..

[20] Venkatesulu B., Thomas M.J. (2010). Corona aging studies on silicone rubber nanocomposites. IEEE Trans. Dielectr. Electr. Insul..

[21] Zeng S., Li W., Peng Y., Zhang Y., Zhang G. (2023). Mechanism of accelerated deterioration of high-temperature vulcanized silicone rubber under multi-factor aging tests considering temperature cycling. Polymers.

[22] Chen C., Jia Z., Ye W., Guan Z., Li Y. (2017). Thermo-oxidative aging analysis of HTV silicone rubber used for outdoor insulation. IEEE Trans. Dielectr. Electr. Insul..

[23] Shaik M.G., Karuppaiyan V. (2019). Investigation of surface degradation of aged high-temperature vulcanized silicone rubber insulators. Energies.

[24] Lu M., Zeng S., Gao C., Liu Y., Yan X., Liu Z., Zhang G. (2025). Aging Analysis of HTV Silicone Rubber Under Coupled Corona Discharge, Humidity and Cyclic Thermal Conditions. Electronics.

[25] (2021). Nanotechnologies—Performance Measurement for Surface Topography Analysis Using Scanning Electron Microscopy.

[26] (2011). Microbeam Analysis—Quantitative Analysis Using Energy-Dispersive Spectrometry (EDS) for Elements with an Atomic Number of 11 (Na) or Above.

[27] (2022). Adhesives—T-Peel Test for Flexible-to-Flexible Bonded Assemblies.

[28] (2023). Plastics—Film and Sheeting—Determination of Water Contact Angle of Matte Film.

[29] (2010). Rubber, Vulcanized or Thermoplastic—Determination of Indentation Hardness—Part 1: Durometer Method (Shore Hardness).

[30] (1969). Recommended Methods for the Determination of the Permittivity and Dielectric Dissipation Factor of Electrical Insulating Materials at Power, Audio and Radio Frequencies Including Meter Wavelengths.

[31] Lin Y., Liu Y.H., Wu K.N., Wang L.M., Ding L.J. (2021). Dynamic electrical conductivity induced by isothermal crystallization in aluminum hydroxide-filled silicone rubber. Appl. Phys. Lett..

